# Serum vitamin D and vitamin D-binding protein levels in mother-neonate pairs during the lactation period

**DOI:** 10.1186/s13052-018-0448-2

**Published:** 2018-01-22

**Authors:** Hakan Doneray, Remziye Seda Yesilcibik, Esra Laloglu, Metin Ingec, Zerrin Orbak

**Affiliations:** 1 Department of Pediatric Endocrinology, Faculty of Medicine, Ataturk University, 25240 Erzurum, Turkey; 2 Department of Pediatrics, Faculty of Medicine, Ataturk University, Erzurum, Turkey; 3 Department of Biochemistry, Erzurum Public Health Laboratory, Erzurum, Turkey; 4 Department of Obstetrics and Gynecology, Faculty of Medicine, Ataturk University, Erzurum, Turkey

**Keywords:** Vitamin D, Vitamin D-binding protein, Lactation, Neonates

## Abstract

**Background:**

To determine longitudinally the relationship between serum 25-hydroxyvitamin D (vitamin D) and vitamin D-binding protein (DBP) levels in mother-neonate pairs and evaluate the efficiency of prophylactic vitamin D on lactation days 45–60.

**Methods:**

Mother-neonate pairs whose serum calcium (Ca), phosphorus (P), magnesium (Mg), alkaline phosphatase (ALP), and parathyroid hormone (PTH) levels were in normal ranges on postpartum/postnatal days 5–10 were classified into two groups by their serum vitamin D concentrations (Group A: < 10 ng/ml and Group B: > 20 ng/ml). Both maternal and neonatal Ca, P, Mg, ALP, and PTH concentrations in group A and B were not different. Maternal and neonatal serum DBP levels were measured in two groups. The mother-neonate pairs in both groups were given 400 IU/d vitamin D orally. The same biochemical markers in group A were remeasured on days 45–60 of the lactation period.

**Results:**

In group A, the mean maternal and neonatal vitamin D levels on postpartum/postnatal days 5–10 were significantly lower and the DBP levels were significantly higher than those in group B (*P* = 0.000; *P* = 0.000 and *P* = 0.04; *P* = 0.004, respectively). On lactation days 45–60, the maternal and neonatal DBP concentrations were not different from those on postpartum/postnatal days 5–10. However, the maternal and neonatal vitamin D levels were significantly increased (*P* = 0.000 and *P* = 0.000, respectively), while the neonatal PTH concentrations were significantly decreased (*P* = 0.000). The maternal and neonatal vitamin D concentrations were negatively correlated with their DBP concentrations (*P* = 0.048 and *P* = 0.002, respectively).

**Conclusion:**

High maternal and neonatal DBP levels may lead to an incorrect low estimate of the true Vitamin D concentration. In this case, only prophylactic vitamin D (400 IU/d) is indicated for mothers and their infants.

## Background

There are a growing number of reports on the high prevalence of vitamin D deficiency in pregnant women and their neonates in many developed and developing countries from areas of different sun exposure [1]. In those studies, the prevalence of vitamin D deficiency ranged from 18% to 84% [1]. However, those figures are usually only based on serum 25-hydroxyvitamin D (vitamin D) concentration, regardless of serum calcium (Ca), phosphorus (P), magnesium (Mg), alkaline phosphatase (ALP), and parathyroid hormone (PTH) levels, in both pregnant women and their neonates. For this reason, they may not reflect true vitamin D status.

Vitamin D-binding protein (DBP) is a multifunctional and highly expressed plasma protein. One of the major functions of DBP is to bind and transport vitamin D, the major circulating metabolite, and 1,25-dihydroxyvitamin D (active vitamin D) [2]. Many physiological and pathological factors can affect plasma DBP levels [3]. Pregnancy has been shown to be associated with an increase in serum DBP level. This situation results from the stimulation of DBP synthesis by estrogen [3]. The high DBP concentration under estrogenic effects in pregnancy may be responsible for low vitamin D concentrations in both pregnant women and their neonates because it binds to vitamin D. To our knowledge, no previous longitudinal study has investigated the relationship between serum vitamin D and DBP in mothers and their neonates during the lactation period.

### Aim of the study

We hypothesized that high maternal and neonatal DBP levels may lead to an incorrect low estimate of the true Vitamin D level and that a prophylactic vitamin D dose (400 IU/d) would be sufficient for these mothers and their infants during the early lactation period. Therefore, the goal of the present study was to assess the relationship between serum vitamin D and DBP levels in both mothers and their neonates and evaluate the effectiveness of prophylactic vitamin D at lactation days 45–60.

## Methods

### Subjects

The University of Ataturk, Faculty of Medicine Ethics Committee approved the study. Written informed consent was obtained from all participants. The study was longitudinal and was conducted in winter and spring months between 2013 and 2016. Serum vitamin D and DBP concentrations of mothers and their neonates were investigated during the first 60 days of the lactation period. First, the mothers were selected from Department of Obstetrics and Gynaecology, Faculty of Medicine, Ataturk University, Erzurum, Turkey, which offers tertiary healthcare for a population of more than 4.000.000 individuals. Exclusion criteria for the mothers were multiple gestation, different ethnicity, delivery within the previous 2 years, pre-existing maternal disease, pre-eclampsia, BMI > 35.0 at enrolment, hypertension, smoking, hormonal or drug therapy, and lactation failure. Second, the neonates of the mothers that met the study criteria were evaluated. Inclusion criteria for neonates were term deliveries with appropriate for gestational age (AGA) after uncomplicated pregnancies. Neonates classified as large for gestational age (LGA) or small for gestational age (SGA) and having any fetal malformation, supplementation with formula, and failure for visit 2 were excluded from the study together with their mothers. Then, serum Ca, P, Mg, ALP, PTH and vitamin D concentrations in the mothers selected for the study were measured on postpartum days 5–10. The mothers having normal serum Ca (8.8–10.8 mg/dl), P (2.7–4.2 mg/dl), Mg (1.2–1.6 mg/dl), ALP (130–560 IU/l), and PTH (1–55 pg/ml) were classified into two groups by their serum vitamin D concentration (Group A: < 10 ng/ml and Group B: > 20 ng/ml), whereas those whose serum vitamin D concentration was 10–20 ng/ml were excluded from the study. Finally, the same biochemical markers in the neonates of the mothers included in the study were measured on postnatal days 5–10. Among the neonates having normal serum Ca (7.6–11.2 mg/dl), P (4.8–8.2 mg/dl), Mg (1.2–1.6 mg/dl), ALP (140–450 IU/l), and PTH (1–43 pg/ml), those having similar vitamin D concentrations as their mothers were included the study, whereas those whose serum vitamin D concentration was 10–20 ng/ml were excluded from the study together with their mothers. The mean maternal and neonatal serum Ca, P, Mg, ALP, and PTH concentrations in group A and B were not significantly different (Tables 1 and 2). Two hundred and 53 mothers were originally selected but 99 of them were excluded from the study on the basis of their clinical and laboratory findings. Of the remaining 154 mother-neonate pairs, 53 neonates having exclusion criteria were excluded from the study together with their mothers. Remaining 101 mother-neonate pairs met the study criteria. However, 41 neonates were excluded from the study for the following reasons: 10 did not come to visit 2 during the follow-up period, 10 had to use formula, 14 stopped vitamin D prophylaxis and 7 had a serum vitamin D concentration that did not fall into the same group of that of their mothers. Therefore, the study was completed in 60 mother-neonate pairs: 30 in group A and 30 in group B. All mothers and their neonates were given an oral prophylactic vitamin D supplement of 400 IU/d because serum vitamin D itself is not sufficient to diagnose vitamin D deficiency, regardless of serum Ca, P, Mg, ALP, and PTH levels. Serum Ca, P, Mg, ALP, PTH and vitamin D concentrations in every mother-neonate pair in Group A were remeasured on any day between the 45th and 60th days of the lactation period (visit 2). The blood samples (2 ml) of mothers and their neonates were obtained from both groups at the beginning of the study and from only group A at visit 2. The samples were placed into a plain evacuated glass tube. The blood samples were centrifuged at 3.500 rpm for 5 min at 4 °C. Sera were pipetted into Eppendorf tubes and stored at − 80 °C for DBP analysis.

**Table 1:** Maternal serum concentrations of Ca, P, Mg, ALP, PTH, vitamin D, and DBP

	Group A (GA)	Group B (GB)	Visit 2^a^ (V2)	*P-value*	
				GA-GB	GA-V2
Ca (mg/dl)	8.98 ± 0.25	8.99 ± 0.20	9.56 ± 0.52	0.82	0.000
P (mg/dl)	3.35 ± 0.35	3.240 ± 0.42	3.53 ± 0.51	0.27	0.303
Mg (mg/dl)	2.02 ± 0.24	2.02 ± 0.17	2.20 ± 0.26	0.97	0.08
ALP (IU/l)	152.97 ± 33.04	159.67 ± 31.46	152.59 ± 37.08	0.45	0.89
PTH (pg/ml)	30.87 ± 11.12	27.56 ± 11.52	27.76 ± 12.04	0.26	0.13
Vitamin D (ng/ml)	8.91 ± 1.52	29.18 ± 6.21	31.28 ± 8.53	0.000	0.000
DBP ((μg/l)	0.51 ± 0.65	0.25 ± 0.28	0.56 ± 0.54	0.04	0.86

Data are presented as mean ± SD

^a^Any day between 45th and 60th days of the lactation period

**Table 2:** Neonatal serum concentrations of Ca, P, Mg, ALP, PTH, vitamin D, and DBP

	Group A (GA)	Group B (GB)	Visit 2^a^ (V2)	*P-value*	
				GA-GB	GA-V2
Ca (mg/dl)	9.25 ± 0.86	9.69 ± 0.59	10.09 ± 0.59	0.057	0.000
P (mg/dl)	5.93 ± 0.93	5.57 ± 0.66	5.94 ± 0.79	0.08	0.97
Mg (mg/dl)	2.12 ± 0.31	2.26 ± 0.32	2.18 ± 0.43	0.089	0.23
ALP (IU/l)	270.03 ± 81.36	288.60 ± 70.63	287.90 ± 89.70	0.34	0.39
PTH (pg/ml)	31.06 ± 9.98	28.65 ± 9.80	25.70 ± 13.01	0.35	0.000
Vitamin D (ng/ml)	9.07 ± 1.19	30.99 ± 8.12	37.74 ± 10.17	0.000	0.000
DBP ((μg/l)	0.69 ± 0.46	0.33 ± 0.45	0.71 ± 0.54	0.004	0.86

Data are presented as mean ± SD

^a^Any day between 45th and 60th days of the lactation period in group A

Anthropometric indexes and the gestational week of infants were recorded. Weight was measured using an electronic scale (Seca Model 770, Hamburg, Germany). Length (±0.1 cm) was measured using a body-length measurer by a pediatrician. Head circumference was measured by a tape measure.

In addition to maternal age and clothing style (covered: Muslim style clothing and uncovered), the duration and the dose of the vitamin D supplementation and the duration of exposure to sunlight during the gestational period were recorded based on self-assessment of the mothers.

### Biochemical analysis

The serum samples were maintained in a refrigerator overnight with the purpose of thawing before analysis. Serum Ca, P, Mg, and ALP levels were determined by spectrophotometric methods, using the Beckman Coulter- AU5800 chemistry analyzer. Serum PTH and vitamin D levels were measured by the immunoassay method, using the Beckman Coulter-DXI800 analyzer. We used a human vitamin D binding protein kit (Human Vitamin D-binding protein, DBP ELISA Kit, SunLong Biotech Co.,LTD, lot number 201610). The limit of sensitivity for the kit was 0.01 μg/ml. The intraassay and interassay coefficients of variation were < 10% and < 12%, respectively.

### Statistical analysis

The total sample size for the study was calculated by a formula below.

$$ \mathrm{N}=\frac{\left(\mathrm{r}+1\right){\left({\mathrm{Z}}_{\upalpha /2}+{\mathrm{Z}}_{1\hbox{-} \upbeta}\right)}^2\kern0.5em {\updelta}^2}{{\mathrm{rd}}^2} $$

r is a ratio (n1/n2) of sample sizes in Group A (n1) and B (n2). We planned that group A and B had equal sample size. Thus, *r* = 1. Z_α/2_ and Z_1-β_ are 1.96 for 5% level of significance and 1.28 for 90% of statistical power, respectively. δ and d represent the pooled standard deviation and difference of the mean for DBP, respectively. According to that formula, the total sample size for each group with 90% of statistical power and 5% level of significance was calculated as 29.

All the calculations were made using SPSS (version 15.0 for Windows). The Kolmogorov-Smirnov test was used for normality. The differences between group A and group B were examined with Student’s t test and Chi square test. Longitudinal changes in the serum parameters in group A were analyzed with the paired t test. Correlations between two variables were tested by Pearson’s correlation co-efficient. The results were expressed as the means ± SD, and statistical significance was set at *P* < 0.05.

## Results

The maternal mean age, clothing style, and vitamin D supplementation dose during pregnancy in group A and B were not different, whereas the duration of the vitamin D supplementation and exposure to sunlight in group A were significantly lower than those in group B (*P* = 0.001 and *P* = 0.02, respectively) (Table 3). In group A, the mean maternal serum vitamin D level was significantly lower and the DBP level was significantly higher than in group B (*P* = 0.000 and *P* = 0.04, respectively) (Table 1). The mean maternal serum P, Mg, ALP, PTH, and DBP concentrations on lactation days 5–10 in group A were not different from those on lactation days 45–60, whereas serum Ca and vitamin D concentrations increased significantly (*P* = 0.000 and *P* = 0.000, respectively) (Table 1).

**Table 3:** Maternal characteristics during pregnancy

	Group A	Group B	*P-value*
Age (yr)	31.17 ± 5.29	33.47 ± 5.22	0.096
Clothing style (covered/ uncovered)	17/13	7/23	0.085
Vitamin D supplementation dose (IU/d)	533.33 ± 125.21	585.22 ± 132.41	0.48
Duration of vitamin D supplementation (wk)	10.07 ± 3.34	12.67 ± 1.98	0.001
Exposure to sunlight (h/d)	0.7 ± 0.28	0.88 ± 0.31	0.02

Data are presented as mean ± SD

The gestational age and neonatal anthropometric measurements at birth in group A and B did not differ significantly (Table 4). In group A, however, the mean neonatal serum vitamin D level was significantly lower, while the DBP level was significantly higher (*P* = 0.000 and *P* = 0.004, respectively) (Table 2). In group A, the mean neonatal serum P, Mg, ALP, and DBP concentrations on lactation days 5–10 were not significantly different from those on lactation days 45–60. However, the mean neonatal serum Ca and vitamin D concentrations increased significantly, while PTH concentration decreased significantly (*P* = 0.000, *P* = 0.000, and *P* = 0.000, respectively) (Table 2).

**Table 4:** Neonatal characteristics at birth

	Group A	Group B	*P-value*
Gestational age (wk)	38.47 ± 0.85	38.32 ± 078	0.48
Head circumference (cm)	35.44 ± 0.76	35.53 ± 0.90	0.68
Length (cm)	51.70 ± 2.54	51.61 ± 2.13	0.89
Body weight (g)	3178.3 ± 339.5	3219.6 ± 372.9	0.65

Data are presented as mean ± SD

**Fig. 1:**
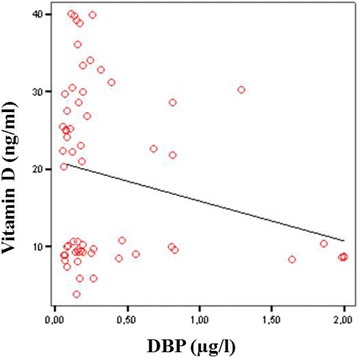
Negative correlation between maternal vitamin D and DBP concentration (*r* = − 0.239, *P* = 0.048)

**Fig. 2:**
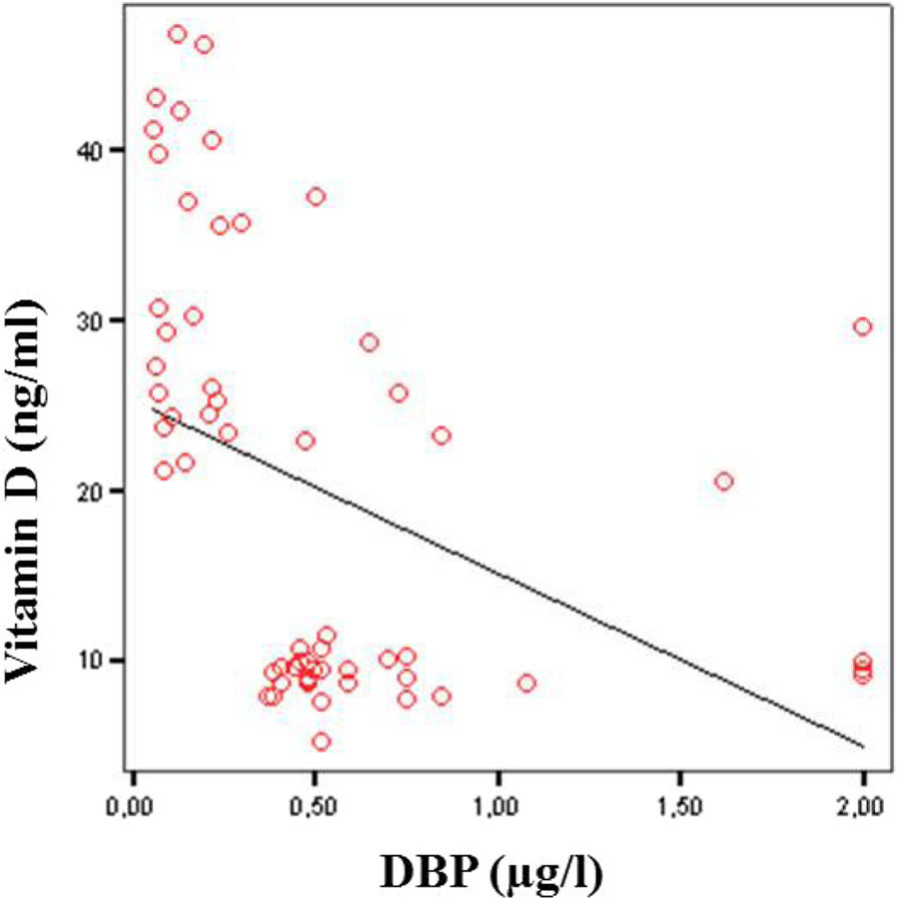
Negative correlation between neonatal vitamin D and DBP concentration (*r* = − 0.401, *P* = 0.002)

Taken together, the data show that the maternal and the neonatal vitamin D concentrations were negatively correlated with the maternal and the neonatal DBP concentrations (*r* = − 0.239, *P* = 0.048 and *r* = − 0.401, *P* = 0.002) (Figs. 1 and 2).

## Discussion

Although there is no consensus on serum vitamin D concentration in children and adults, serum vitamin D levels of < 10 ng/ml (25 nmol/l) and of 20–50 ng/ml (50–125 nmol/l) are considered to represent vitamin D deficiency and sufficiency, respectively [4–7]. According to that information, in this study, group A and group B represent vitamin D deficiency and vitamin D sufficiency, respectively. Because vitamin D primarily controls dietary calcium absorption, serum Ca level, even with a mild insufficiency of vitamin D, is compensated by an increase in serum PTH level, which in turn is associated with high ALP and low serum P levels [7]. Therefore, the relationship between serum PTH and vitamin D is used in many studies to define the normal range of serum vitamin D [8–11]. The sufficient serum vitamin D level is defined as the serum vitamin D concentration that can maintain a serum PTH concentration in a normal range [7]. Because Mg is necessary for PTH secretion, the serum Mg level should also be normal [12]. In our study, the maternal and the neonatal serum Mg levels between group A and B were not different. The serum maternal and neonatal vitamin D levels in group A were significantly lower compared to group B. However, there were no expected changes in group A in terms of serum Ca, P, ALP, and PTH levels. This finding suggests that the vitamin D deficiency in group A was not a true deficiency. In other words, the bioactivity of vitamin D in group A is actually sufficient at the target tissue level. The free hormone hypothesis states that protein-bound hormones are relatively inactive, while hormones not bound to binding proteins have biological activity [13]. The majority (85–90%) of vitamin D and active vitamin D in circulation is tightly bound to DBP, whereas a smaller amount (10–15%) is bound to albumin. Less than 1% of vitamin D in the circulation exists in a free and unbound form [14, 15]. For the exact measurement of serum vitamin D, vitamin D should be separated from albumin and DBP. However, the displacement of vitamin D from DBP is a major challenge. The organic solvents used for this process are not compatible with most immunoassays or protein-binding assays [16–18]. A clinical chemistry study demonstrated the analytical problems in available vitamin D assays [19]. In that study, five automated immunoassays (Architect, Centaur, iSYS, Liaison, and Elecsys), one RIA (Diasorin), and an ID-XLC-MS/MS method were tested for vitamin D measurement in the same plasma samples of 51 healthy individuals, 52 pregnant women, 50 hemodialysis patients, and 50 intensive care patients. The DBP level was also measured in the same plasma samples by ELISA. With the ID-XLC -MS/MS method accepted as a gold standard, Architect, Centaur, iSYS, and Liaison automated immunoassays show an inverse relationship between serum DBP concentrations and deviations of measured vitamin D concentrations from ID-XLC -MS/MS. This finding suggests that these automated assays cannot extract all vitamin D from the DBP in sera. Such incomplete extraction may suggest an incorrectly low vitamin D concentration. In the present study, we used a Beckman Coulter automated immunoassay to measure serum vitamin D level. This assay may be responsible for incorrectly low serum vitamin D concentrations, especially in the presence of high serum DBP concentrations, because it is a similar immunoassay method to those in the study mentioned above. In this study, the negative correlations between serum DBP and vitamin D in both mothers and neonates supports this hypothesis.

It has been shown that ethnicity and DBP genotype, age and gender, pregnancy and estrogen status, obesity, liver and renal diseases, diabetes, primary hyperparathyroidism, cancers, inflammation, and some therapeutic procedures such as plasma exchange and peritoneal dialysis may affect serum DBP and vitamin D concentrations [20]. The DBP level in pregnancy increases as a result of the stimulation of DBP synthesis by estrogen [3, 21]. In our study, the mothers and their neonates did not have any disorders. The ethnicity and age of the mothers were the same. Therefore, it can be said that estrogen in both mothers and their neonates is a major determinant of serum DBP concentration. In our study, however, we found that the maternal and neonatal serum DBP levels in group A and B were different. This finding suggests that some other factors apart from estrogen in pregnancy may affect serum DBP level.

In the follow-up of group A, both maternal and neonatal serum DBP concentrations were not different from those of lactation days 5–10, while serum vitamin D concentrations increased significantly. Vitamin D supplementation (400 IU/d) might have contributed to these higher concentrations. In addition, because vitamin D supplementation may just maintain the serum vitamin D concentration at a similar level, we can also speculate that the increase in serum vitamin D levels may be related to the release of vitamin D from DBP overtime.

A high prevalence of subclinical vitamin D deficiency in pregnant women and neonates has been reported in many countries. In a review, 18% of pregnant women in the United Kingdom, 25% in the United Arab Emirates, 80% in Iran, 42% in northern India, 61% in New Zealand and 60–84% of pregnant non-Western women in the Netherlands have been shown to have serum vitamin D concentrations < 10 ng/ml [1]. Similarly, studies from the United Arab Emirates, Iran, India, the United Kingdom, Greece and the US demonstrate a high prevalence of vitamin D deficiency in mother-infant pairs at birth [1]. In those studies, the prevalence of vitamin D deficiency is only based on serum vitamin D levels measured with immunoassays, regardless of serum Ca, P, Mg, ALP and PTH levels. The findings of our study suggest that even if serum vitamin D levels in a mother-neonate pair are lower than 10 ng/ml, this finding is not sufficient to diagnose vitamin D deficiency, regardless of serum Ca, P, Mg, ALP and PTH levels. Accordingly, it can also be said that the real prevalence of vitamin D deficiency in these populations may be lower.

Our study has several limitations: 1) The number of subjects was relatively small and therefore, the power of the study is relatively limited; 2) All biochemical parameters in group B were in normal range. Therefore, they were measured only once for ethical reasons. Thus, the change in the biochemical parameters with time could not evaluated for group B; 3). We used an automated immunoassay method to measure serum vitamin D levels since only mothers and their infants whose serum Ca, P, Mg, ALP and PTH levels were normal were included in the study. However, it should be emphasized that, due to the analytical problem in available vitamin D assay, the serum vitamin D levels could also be measured by LC-MS/MS method.

## Conclusion

This is the first study that investigated the relationship between maternal and neonatal serum vitamin D and DBP during lactation. The findings of our study suggest that the high maternal and the neonatal serum DBP levels may be associated with falsely low vitamin D concentrations based on the normal serum Ca, P, Mg, ALP and PTH levels. Even if maternal and neonatal serum vitamin D concentrations are consistent with each other in terms of low serum vitamin D levels (< 10 ng/ml), this finding alone is not sufficient to diagnose vitamin D deficiency, without taking into consideration the serum Ca, P, Mg, ALP, and PTH levels. The lack of expected changes in serum Ca, P, ALP, and PTH levels together with low concentrations of vitamin D deficiency and a normal serum Mg may result from the measurement method and/or from high DBP levels, and may not reflect a true vitamin D deficiency. In this case, only prophylactic vitamin D (400 IU/d) is indicated for mothers and their infants.

## References

[1] Dawodu A, Wagner CL (2007). Mother-child vitamin D deficiency: an international perspective. Arch Dis Child.

[2] White P, Cooke N (2000). The multifunctional properties and characteristics of vitamin D-binding protein. Trends Endocrinol Metab.

[3] Møller UK, Streym S, Heickendorff L, Mosekilde L, Rejnmark L (2012). Effects of 25OHD concentrations on chances of pregnancy and pregnancy outcomes: a cohort study in healthy Danish women. Eur J Clin Nutr.

[4] Rovner AJ, O'Brien KO (2008). Hypovitaminosis D among healthy children in the United States: a review of the current evidence. Arch Pediatr Adolesc Med.

[5] Esposito S, Lelii M (2015). Vitamin D and respiratory tract infections in childhood. BMC Infect Dis.

[6] Wimalawansa SJ (2012). Vitamin D in the new millennium. Curr Osteoporos Rep.

[7] Saliba W, Barnett O, Rennert HS, Lavi I, Rennert G (2011). The relationship between serum 25(OH)D and parathyroid hormone levels. Am J Med.

[8] Okazaki R, Sugimoto T, Kaji H, Fujii Y, Shiraki M, Inoue D, Endo I, Okano T, Hirota T, Kurahashi I, Matsumoto T (2011). Vitamin D insufficiency defined by serum 25-hydroxyvitamin D and parathyroid hormone before and after oral vitamin D_3_ load in Japanese subjects. J Bone Miner Metab.

[9] Holick MF, Siris ES, Binkley N, Beard MK, Khan A, Katzer JT, Petruschke RA, Chen E, de Papp AE (2005). Prevalence of vitamin D inadequacy among postmenopausal north American women receiving osteoporosis therapy. J Clin Endocrinol Metab.

[10] Carnevale V, Nieddu L, Romagnoli E, Battista C, Mascia ML, Chiodini I, Eller-Vainicher C, Frusciante V, Santini SA, La Porta M, Minisola S, Scillitani A (2010). Regulation of PTH secretion by 25-hydroxyvitamin D and ionized calcium depends on vitamin D status: a study in a large cohort of healthy subjects. Bone.

[11] Lips P, Duong T, Oleksik A, Black D, Cummings S, Cox D, Nickelsen T (2001). A global study of vitamin D status and parathyroid function in postmenopausal women with osteoporosis: baseline data from the multiple outcomes of raloxifene evaluation clinical trial. J Clin Endocrinol Metab.

[12] Vetter T, Lohse MJ (2002). Magnesium and the parathyroid. Curr Opin Nephrol Hypertens.

[13] Mendel CM (1989). The free hormone hypothesis: a physiologically based mathematical model. Endocr Rev.

[14] Bikle DD, Siiteri PK, Ryzen E, Haddad JG (1985). Serum protein binding of 1,25-dihydroxyvitamin D: a reevaluation by direct measurement of free metabolite levels. J Clin Endocrinol Metab.

[15] Chun RF, Peercy BE, Orwoll ES, Nielson CM, Adams JS, Hewison M (2014). Vitamin D and DBP: the free hormone hypothesis revisited. J Steroid Biochem Mol Biol.

[16] Vogeser M (2010). Quantification of circulating 25-hydroxyvitamin D by liquid chromatography tandem mass spectrometry. J Steroid Biochem Mol Biol.

[17] Wallace AM, Gibson S, de la Hunty A, Lamberg-Allardt C, Ashwell M (2010). Measurement of 25-hydroxyvitamin D in the clinical laboratory: current procedures, performance characteristics and limitations. Steroids.

[18] Beastall G, Rainbow S (2008). Vitamin D reinvented: implications for clinical chemistry. Clin Chem.

[19] Heijboer AC, Blankenstein MA, Kema IP, Buijs MM (2012). Accuracy of 6 routine 25-hydroxyvitamin D assays: influence of vitamin D binding protein concentration. Clin Chem.

[20] Yousefzadeh P, Shapses SA, Wang X (2014). Vitamin D binding protein impact on 25-Hydroxyvitamin D levels under different physiologic and pathologic conditions. Int J Endocrinol.

[21] Sinotte M, Diorio C, Bérubé S, Pollak M, Brisson J (2009). Genetic polymorphisms of the vitamin D binding protein and plasma concentrations of 25-hydroxyvitamin D in premenopausal women. Am J Clin Nutr.

